# Supplementary material for: Attenuation Correction of Long Axial Field-of-View Positron Emission Tomography Using Synthetic Computed Tomography Derived from the Emission Data: Application to Low-Count Studies and Multiple Tracers
Source: Diagnostics (Basel). 2023 Dec 14;13(24):3661. doi: 10.3390/diagnostics13243661 (PMC10742516; doi:10.3390/diagnostics13243661)
Supplement: Supplementary file 1 [file diagnostics-13-03661-s001.zip › diagnostics-2714932-supplementary.pdf]

diagnostics

MDPI

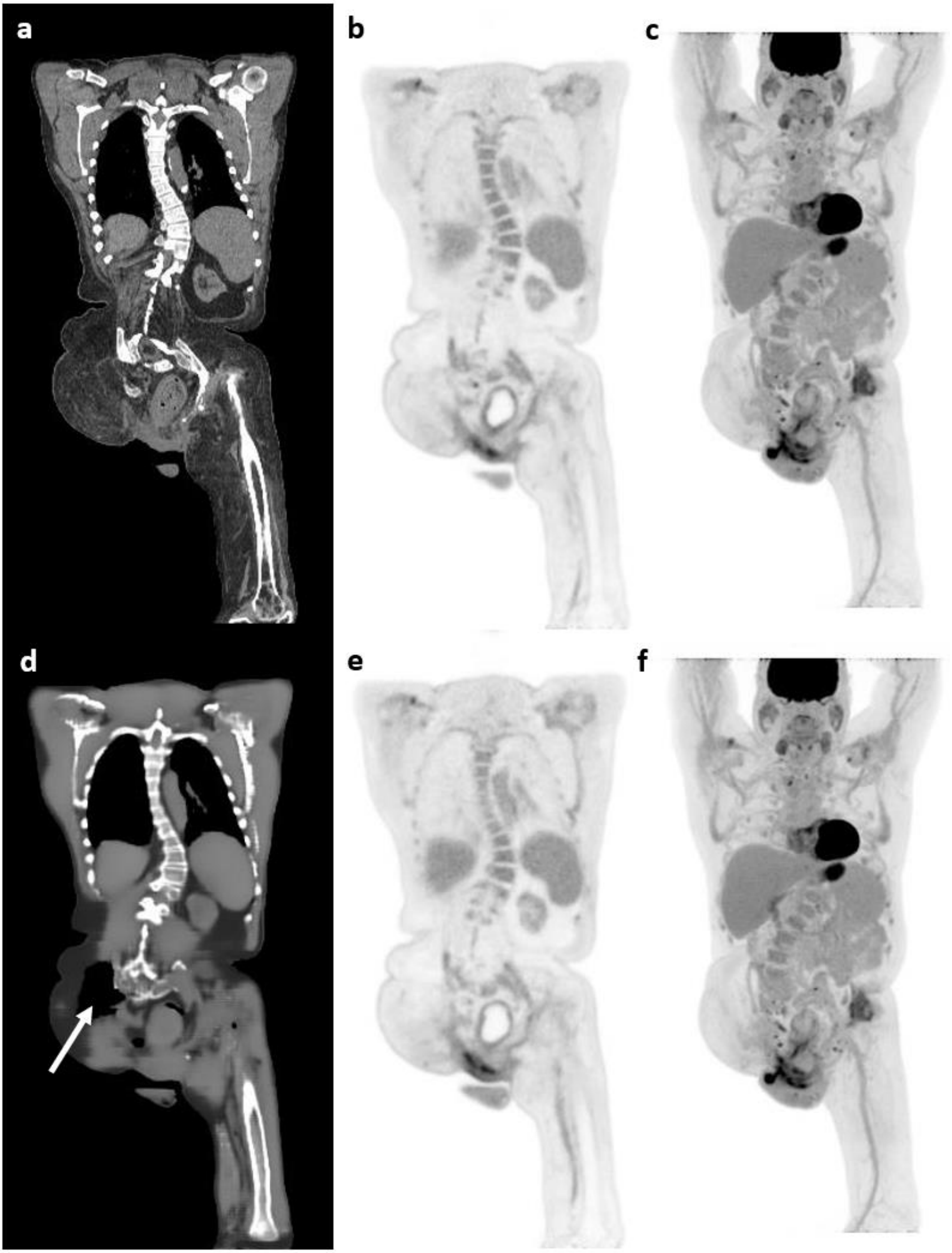

Figure S1: Sample patient illustration with abnormal anatomy. Left column show coronal section of CT (a) and sCT (d). Center column PET (b) and sPET (e) and right column MIP PET (c) and MIP sPET (f). Good recovery is achieved in the upper thorax/lung region including spine and aorta. The abdominal area is more challenging, note the air pocket placed in the patient right side (arrow).
